# Use of psychiatric drugs in Dermatology^☆^^☆☆^

**DOI:** 10.1016/j.abd.2019.12.002

**Published:** 2020-02-18

**Authors:** Magda Blessmann Weber, Júlia Kanaan Recuero, Camila Saraiva Almeida

**Affiliations:** Dermatology Service, Universidade Federal de Ciências da Saúde de Porto Alegre, Porto Alegre, RS, Brazil

**Keywords:** Antidepressive agents, Dermatology, Psychopharmacology, Psychosomatic medicine, Psychotropic drugs

## Abstract

Patients with psychocutaneous disorders often refuse psychiatric intervention in their first consultations, leaving initial management to the dermatologist. The use of psychotropic agents in dermatological practice, represented by antidepressants, antipsychotics, anxiolytics, and mood stabilizers, should be indicated so that patients receive the most suitable treatment rapidly. It is important for dermatologists to be familiar with the most commonly used drugs for the best management of psychiatric symptoms associated with dermatoses, as well as to manage dermatologic symptoms triggered by psychiatric disorders.

## Introduction

The prevalence of psychiatric comorbidities is higher and more frequent in dermatological patients than in the general population.^1^ It is estimated that 25–30% of patients have some mental disorder or emotional problem, which may represent the cause, predisposition, or aggravation of the skin condition.^1^, ^2^ Psychodermatology studies skin diseases resulting from the skin-mind interaction, through its union with psychiatry.^3^ It includes skin manifestations resulting from or worsened by psychological factors and the assessment of mental and social damage resulting from these dermatoses. The management of psychodermatoses is essential in the field of dermatology, since dermatologists are responsible for most outpatient care due to psychocutaneous complaints.^4^ Moreover, many of these patients refuse psychiatric intervention – either due to the stigma associated with mental illnesses or the non-acceptance of the psychological component in their skin condition, leaving the management to the dermatologist alone.^5^ When there is resistance to psychiatric treatment, the dermatologist should support the patient from a non-judgmental position, prescribe the indicated psychotropic medication, and encourage evaluation with a psychiatrist as a complement and not as a substitute for the therapeutic relationship.

The associated use of psychotropic drugs, such as antidepressants, antipsychotics, anxiolytics, and mood stabilizers, is essential for these patients, as their skin lesions can worsen if the underlying psychopathologies are not treated. Thus, knowledge and confidence in prescribing the most used psychotropics aid the management of the psychiatric symptoms associated with dermatoses, as well as the management of dermatological symptoms triggered by psychiatric syndromes.

Clinical situations in which knowledge of psychotropics is required of the dermatologist^2^:

1. Management of dermatological symptoms associated with psychiatric disorders;
2. Management of psychiatric symptoms associated with dermatological conditions, such as social phobia in patients with vitiligo;
3. Management of adverse effects associated with the use of psychotropic drugs;
4. Management of other pharmacological effects of these medications, such as the anticholinergic and antihistamine effects of antidepressants and antipsychotics.

## Classification of psychodermatoses

Psychodermatoses can be classified into six categories^6^:

1. Psychophysiological disorders: Primary dermatoses that are exacerbated by emotional factors and stress. Examples: psoriasis and atopic dermatitis;
2. Primary psychiatric disorders: Primary psychiatric diseases that present self-inflicted skin manifestations as a secondary manifestation of the psychiatric illness. Examples: trichotillomania, parasitic delirium, dermatitis artefacta, and neurotic excoriations;
3. Secondary psychiatric disorders: Psychiatric illnesses that arise as a result of the psychosocial impact of existing dermatoses. Examples: social phobia, depression that arises from psoriasis, and alopecia areata;
4. Sensitive skin disease: Psychogenic symptoms, such as pruritus or burning, without evidence of skin disease or other medical condition. Examples: vulvodynia and glossodynia;
5. Alterations caused by the use of psychoactive drugs for dermatological treatment. Examples: pruritus, rash, and Stevens–Johnson syndrome;
6. Multifactorial diseases: Conditions in which psychoneuroimmunological factors trigger or aggravate skin conditions. Examples: atopic dermatitis, psoriasis, alopecia areata, chronic pruritus.

Most patients with psychodermatoses are classified among the following psychiatric diagnoses^7^: depressive disorders; anxiety disorders; psychotic disorders and delirium disorders; obsessive–compulsive disorder; and impulse control disorders.

Although dermatologists do not have specific training to perform psychiatric diagnoses, a solid doctor-patient relationship, developed over several consultations, can assist them in identifying underlying psychiatric illnesses. Thereafter, they should be able to prescribe the psychotropic drugs indicated for the specific psychiatric illness.^7^, ^8^

## Antidepressants

The use of antidepressants is based on the monoaminergic theory of depression, in which deficiencies in serotonin, norepinephrine, and/or dopamine are implicated in the genesis of the disease. Thus, the different classes of antidepressants act to increase these neurotransmitters, either by inhibiting their reuptake, or by inhibiting the enzyme responsible for their degradation (monoamine oxidase inhibitors).^9^ Furthermore, they are also approved for the treatment of anxiety disorders, social phobia, and obsessive–compulsive disorder.

None of the antidepressant classes has been shown to be the most effective in treating depression and none is specifically indicated for each psychodermatologic disease. They reach their therapeutic dose in a period of four to six weeks, but the recommendation is to start with low doses and gradually increase – preferably at least every 14 days. In the absence of a response at the end of the initial six weeks, an alternative drug should be chosen. If a partial improvement in symptoms is observed, the doses should be increased until the ideal dose for each patient, assessed individually, is reached.^10^ The adverse effects are different for each class, and are more often reported with the use of tricyclic antidepressants. While these drugs do not cause dependence, symptoms such as insomnia, nausea, sweating, and sensory disturbances are described after abrupt discontinuation. For withdrawal, the dose should be gradually decreased over several weeks.^11^ Treatment should be maintained for at least six months after a therapeutic response before attempting to withdraw, in order to minimize the risk of recurrence of symptoms.^10^, ^12^

### Selective serotonin reuptake inhibitors^10^

Selective serotonin reuptake inhibitors (SSRIs), listed in table 1, act by selectively inhibiting serotonin reuptake, thereby increasing the availability of this neurotransmitter, responsible for influencing mood, cognition, sleep, appetite, and sexual behavior.^13^ The monoaminergic theory of depression postulates that increasing the availability of serotonin in the synaptic cleft would modulate the improvement of depression symptoms.

**Table 1:** Main types of selective serotonin reuptake inhibitors (SSRI).

Medication	Brand name	Presentation	Initial dose – maximum	Observations
Fluoxetine	Prozac, Daforin	10 and 20 mg tablet/capsule	10–80 mg/day	No monitoring required
		Oral solution 20 mg/mL		Extensive experience in pregnant women
				Long half life
Paroxetine	Paxil, Pondera, Aropax	10, 20, and 30 mg tablet	20–60 mg	No monitoring required
Sertraline	Tolrest, Zoloft, Assert	25, 50, and 100 mg tablet	25–200 mg	Used in patients with liver problems
Fluvoxamine	Luvox, Revoc	50 and 100 mg tablet	50–300 mg	Fractionize dose if > 150 mg/day
Citalopram	Celexa, Procimax	20 and 40 mg tablet	20–60 mg	Recommended in liver disease
				Higher cardiac risk at doses > 40 mg/day
Escitalopram	Lexapro, Reconter	5, 10, and 20 mg tablet	5–20 mg	
		Oral solution 20 mg/mL		

They have a good safety profile and tend to have greater tolerability when compared with tricyclic antidepressants, being the first therapeutic choice for many patients. The most reported adverse effects are gastrointestinal changes (nausea and dyspepsia), insomnia, weight change, and sexual dysfunction, such as anorgasmia and reduced libido.^14^ They can be used by pregnant women; for such patients, those with shorter half-life, such as sertraline and paroxetine, are preferred.^15^

### Tricyclic antidepressants^10^

This is the oldest class of antidepressants, listed in table 2. They act similarly to SSRIs, increasing serotonin and norepinephrine in the synaptic cleft. They have been replaced by SSRIs over the years, due to their more sedative effects and a greater number of other side effects. However, these drugs (especially doxepin) present properties more similar to antihistamines, and are therefore widely used for insomnia and pruritus.^15^, ^16^ They also perform well in patients with pain of neural origin. Generally, the doses needed to treat pain and pruritus tend to be lower than antidepressant doses. Nortriptyline has fewer adverse effects and should be chosen for elderly patients.^11^

**Table 2:** Main types of tricyclic antidepressants.

Medication	Brand name	Presentation	Initial dose – maximum	Observations
Nortriptyline	Pamelor	10, 25, 50, and 75 mg capsules	10–150 mg/day	EKG in women > 40 years and men > 30 years
		Oral solution 2 mg/mL		Safer for the elderly
Amitriptyline	Amytril, Tryptanol	10, 25, and 75 mg tablets	10–150 mg/day	EKG in women > 40 years and men > 30 years
Doxepin	Not available in Brazil	–	100–300 mg/day	Need to be formulated
Clomipramine	Anafranil	10, 25, and 75 mg tablet/dragée	10–250 mg/day	EKG in women > 40 years and men > 30 years
		Injectable solution 25 mg/2 mL		

Among the adverse effects, the literature describes dry mouth, constipation, dizziness, blurred vision, tachycardia, and urinary retention.^17^ They should be used with caution in patients with cardiac conditions, such as conduction disorder. There is an absolute contraindication for their use in patients after a recent episode (up to six weeks) of acute myocardial infarction.^15^ They can be used during pregnancy, although they should not be prescribed in the first trimester.^18^

### Doxepin (Sinequan®)

This tricyclic antidepressant has potent antihistamine properties; in dermatology, it is used in patients with chronic pruritus and urticaria, representing an option to diphenhydramine and hydroxyzine.^12^, ^17^ Furthermore, when in topical formulation (5% cream), it does not cause the side reactions characteristic of oral tricyclic antidepressants.^16^, ^19^ When used orally, the initial dose is 25 mg/day; it can be increased weekly by 10–25 mg, reaching a maximum of 100 mg/day.

Sedation is the main adverse effect, and dose schedule adjustment may be necessary in case of patient complaints. Patients with a history of heart rhythm alterations should undergo an electrocardiogram before starting treatment. If the dose is increased, it is suggested that the test be repeated when the dosage reaches 100 mg/day.^12^ Currently, in Brazil, this medicine is only provided by handling pharmacy.

### Other antidepressants^10^

These antidepressant drugs are listed in table 3.

**Table 3:** Main types of other antidepressants.

Medication	Brand name	Presentation	Initial dose – maximum	Observations
Mirtazapine	Remeron, Zispin, Norset	15, 30, and 45 mg tablet	15–45 mg/day	Sedative
				Weight gain
				Anticholinergic effects (rare)
Bupropion	Wellbutrin, Wellbutrin XL Zetron, Zetron XL	150 and 300 mg tablet	150–300 mg/day	Slow or immediate release
Venlafaxine	Effexor, Zyvifax, Zaredrop	37.5, 50, 75, and 150 mg tablet/capsule	75–300 mg/day	Fractionized dose
		Oral solution 75 mg/mL		
Duloxetine	Velija, Cymbalta	30 and 60 mg tablet/capsule	60 mg/day	–
Trazodone	Donaren	50 and 100 mg tablet	150–600 mg/day	Sedative
				Fractionized doses above 300 mg/day
				Use in hospitalized patients

### Mirtazapine (Remeron®, Zispin®, Norset®)

A tetracyclic antidepressant that acts directly, by increasing the amount of serotonin and norepinephrine. Due to its high potential for sedation and weight gain, it is preferably used in terminal patients.^11^

### Bupropion (Wellbutrin®, Zetron®)

Bupropion is a selective norepinephrine and dopamine reuptake drug. As it has fewer sexual adverse effects and a similar antidepressant capacity, it is preferred in patients with complaints of libido alterations.^12^ Its use should also be considered in patients with sleep disorders.^11^ Dose fractioning is necessary, except in cases of slow-release tablets. It is a generally well-tolerated medication, and its main side effects are insomnia, agitation, headache, constipation, dry mouth, nausea, and tremors. Seizures are rare effects, but they can be observed; therefore, care should be taken when indicating use in patients with a history of epilepsy. Bupropion should also be avoided in patients with a history of alcohol and drug abuse.^12^, ^16^ Its use in pregnancy is not recommended.^18^

### Venlafaxine (Efexor®, Zyvifax®)

Officially released for use in depression and anxiety, venlafaxine appears to act in the reception of serotonin and norepinephrine. The initial recommended dose is 75 mg/day, increasing every two weeks, reaching a maximum dose of 300 mg/day that should be divided into two doses. The most commonly reported adverse effects of this drug are insomnia and anxiety; when used in high doses, blood pressure should be monitored.^14^, ^15^

Regarding the use in pregnancy, despite the lack of studies in pregnant women, experiments with animals have not demonstrated teratogenicity.^18^

## Antipsychotics^10^

Antipsychotics, listed in table 4, are dopamine receptor antagonists, acting mainly by blocking D2 subtype receptors.^20^ They are divided into typical (pimozide, chlorpromazine, and haloperidol) and atypical antipsychotics (risperidone, olanzapine, sulpiride, and quetiapine). Atypical antipsychotics have a lower affinity for D2 receptors, so they have a lower incidence of extrapyramidal effects, such as dystonia and parkinsonism, than typical antipsychotics.

**Table 4:** Main types of antipsychotics.

Medication	Brand name	Presentation	Initial dose – maximum
Pimozide	Orap	1 and 4 mg tablet	2–4 mg/day – 20 mg/day
Risperidone	Risperdal, Riss	1, 2, and 3 mg tablet 1 mg/mL solution	2 mg/day – 8 mg/day
Olanzapine	Zyprexa, Zopix	2.5, 5, and 10 mg tablet	2.5–5 mg/day – 15 mg/day
Quetiapine	Seroquel, Quetrox, Queropax	25, 100, and 200 mg tablet	25–750 mg/day
Aripiprazole	Aristab, Abilify	10, 15, 20, and 30 mg tablet	10–30 mg/day
Ziprasidone	Geodon	40 and 80 mg capsule	40–160 mg/day
Haloperidol	Haldol	1 and 5 mg tablet Oral solution 2 mg/mL Injectable solution 5 mg/mL	0.5–15 mg/day

The main dopaminergic pathways in the central nervous system are the mesocorticolimbic, nigrostriatal, and tuberoinfundibular. Dysfunctions in the mesocorticolimbic pathway are associated with psychosis, schizophrenia, and attention deficit disorder, while dysfunction in the nigrostriatal pathway is related to Parkinson's disease, as well as to motor side effects when used in dopaminergic therapy, including extrapyramidal effects. In turn, dysfunctions in the tuberoinfundibular pathway cause changes in prolactin secretion, which can cause galactorrhea and amenorrhea, as dopamine is responsible for inhibiting prolactin secretion.^13^

Antipsychotics may cause side effects due to binding to other receptors, such as weight gain (histamine receptors), orthostatic hypotension (α-adrenergics), constipation, and xerostomia (muscarinic receptors).^20^ They may also increase the risk of myocardial infarction and transient ischemic events in elderly patients.^10^

In dermatology, antipsychotics can be used mainly in delusional disorders, such as parasitic delirium and dermatitis artefacta.^17^

Haloperidol is the best studied drug in relation to use during pregnancy, being the preferred antipsychotic for these patients.^21^

### Pimozide (Orap®)

This is the first generation antipsychotic most widely used in psychodermatology. It acts as a potent antagonist of the central dopamine receptor. It is indicated to start with 1 mg/day, with a progressive increase every two weeks, until reaching the target dose (2–6 mg/day). This dose should be maintained for at least one month after the improvement of symptoms. Although rare, due to the low dose used for skin diseases, the literature describes some adverse effects, such as akathisia, muscle stiffness, and restlessness. Due to the possibility of altering the QT interval, an electrocardiogram is recommended before treatment in patients with a history of heart diseaset. In young/healthy patients, the need for electrocardiographic examination is still controversial.^16^

### Risperidone (Risperdal®, Riss®)

The main medication used for parasitic delirium, risperidone is a new generation antipsychotic and should be started at a dose of 0.5 mg before bedtime, and should be increased weekly until reaching a maximum dose of 4 mg/day. It can cause hyperprolactinemia and, consequently, galactorrhea, amenorrhea, and sexual dysfunction. Other adverse effects, such as sedation, are uncommon and usually resolve within the first few days. This medication should also be used with caution in patients with a history of abnormal electrocardiogram.^11^, ^22^

### Olanzapina (Zyprexa®, Zopix®)

Olanzapine is recommended in low doses for psychodermatologic diseases. The suggested initial dose is 5–10 mg/day until reaching a target dose of 15 mg/day. Despite being a generally well-tolerated medication, its main adverse effect is weight gain and, consequently, metabolic syndrome and increased cardiovascular risk. As a result, it is necessary to control weight and monitor blood pressure, glucose, and lipids during treatment with this medication.^11^, ^14^, ^17^

### Quetiapine (Seroquel®, Quetrox®, Queropax®)

Quetiapine is well indicated in patients resistant to previous treatments and in elderly patients. In the treatment of non-dermatological psychoses, it is initially prescribed at a dose of 25 mg twice a day and increased to 750 mg/day. However, for dermatological use it is recommended to reduce the starting and maintenance doses. For example, 150 mg divided into two doses, is indicated for the treatment of parasitic delirium.^12^, ^23^

Weight gain, drowsiness and orthostatic hypotension are among the most commonly reported side effects.^11^, ^14^

### Aripiprazole (Aristab®, Abilify®)

Aripiprazole is a new generation antipsychotic, noteworthy due to its low relationship with metabolic disorders and cholinergic effects. It has been used for patients with parasitic delirium and neurotic excoriations, with good results at doses between 2 and 30 mg/day. It usually does not cause changes in weight.^14^

### Ziprasidone (Geodon®)

Similar to aripiprazole, ziprasidone is also a new generation antipsychotic. It differs from the others due to lack of anticholinergic effects and the low propensity to metabolic syndrome, in addition to low incidence of sedative effects. However, it has more risks of causing QT interval alterations than other atypical antipsychotics. It is successfully used in the treatment of patients with parasitic delirium with doses ranging from 20 mg to 80 mg/twice daily.^11^

## Mood stabilizers

Mood stabilizers, such as antiepileptic drugs and lithium, are approved for use in bipolar disorder and epilepsy. Their mechanisms are not completely understood, but they are believed to act on the central nervous system, with the power to control neuronal excitation.^24^

Many of these medications, as mentioned in table 5, are used in neuropathic skin pain and are also effective in the management of chronic pruritus, in addition to other skin sensory disorders, such as self-induced dermatoses. Primarily due to their compulsion control power, they are included in the treatment of dermatoses related to self-excoriation, such as nodular prurigo and lichen simplex chronicus, as well as in self-inflicted lesions, such as trichotillomania and dermatitis artefacta. These medications are known to be teratogenic and should be avoided, if possible, in female patients of childbearing age.^14^

**Table 5:** Main types of mood stabilizers and anticonvulsants.

Medication	Brand name	Presentation	Initial dose – Maximum	Observations
Lithium	Carbolitium	300 and 450 mg tablet	600–2.400 mg/day	Serum lithium level assessment is required
Lamotrigine	Lamitor, Lamictal	25, 50, and 100 mg tablet	50–200 mg/day	SJS/TEN
Carbamazepine	Tegretol, Tegrex, Carmazin, Tegretard	200 and 400 mg tablet	200–1600 mg/day	SJS/TEN (HLA-B1502 allele)
		Oral solution 20 mg/mL		
Sodium valproate	Depakote, Depakene	300 and 500 mg tablet	15–60 mg/kg/day	Hepatotoxicity
		250 mg/5 mL syrup		Pancreatitis
				Teratogenic
Topiramate	Amato, Sigmax	25, 50, and 100 mg tablet	50–200 mg/day	Myopia, closed-angle glaucoma
				Metabolic acidosis
				Cognitive dysfunction
				Suicidal behavior
Pregabalin	Lyrica	75 and 150 mg capsule	150–600 mg/day	Suicidal behavior, convulsion in case of rapid withdrawal, angioedema
Gabapentin	Gabaneurin, Gamibetal, Progresse	300 and 400 mg capsule	900–3600 mg/day	–

### Lithium (Carbolitium®)

Lithium carbonate, used in the treatment of bipolar disorder and severe depression, alters the metabolism of neuronal catecholamines. Serum lithium dosage should be monitored initially every 60–90 days, and this interval can be extended up to six months, depending on the time of use. Blood samples should be collected eight to 12 h after taking the previous dose and before the next dose. It is recommended to maintain serum lithium levels between 0.5 and 1.0 mEq/L, equivalent to a dose of 600–900 mg/day. Signs of toxicity may begin to appear as early as 1.0–1.5 mEq/L. The use of this drug also requires frequent monitoring of renal and thyroid functions. Caution should be used when prescribing lithium, due to its high rate of adverse skin effects, such as worsening or triggering the onset of psoriasis and acne.^11^, ^24^, ^25^ Lithium is indicated for the control of impulsive behavior in patients with trichotillomania, due to its high association with obsessive–compulsive disorder, with doses varying between 900 and 1500 mg.^12^, ^26^ Skin picking patients can also benefit from the use of lithium for impulse control, although there is no dose specified in the literature.^11^

### Lamotrigine (Lamitor®, Lamictal®)

Lamotrigine is a medication used to treat seizures. Pharmacologically, it acts on neuronal sodium channels, mainly by stabilizing membranes and inhibiting the release of glutamate. Its benefits in the treatment of skin picking have been demonstrated,^11^ and the literature suggests the use of doses between 12.5 and 300 mg/day.^11^

### Carbamazepine (Tegretol®, Tegrezin®, Tegretard®, Tegrex®, Carmazin®)

Although its action has not been fully understood, carbamazepine is known to stabilize the hyperexcited nerve membrane. It is indicated for the treatment of epilepsy, bipolar disorder, depression, and pain of neural origin. In dermatology, the use of this medication in post-herpetic neuralgia is noteworthy. In addition to dose fractioning, it is recommended to start with lower doses with progressive increase. The recommended dose for neuropathic pain is 600–1200 mg/day. Adverse effects such as gastrointestinal symptoms, dizziness, and blurred vision are reported, as well as skin effects, reported later in this article. The literature also reports more rare risks, such as agranulocytosis and aplastic anemia.^24^

### Sodium valproate (Depakote®, Depakene®, Zyvalprex®)

The activity of sodium valproate, which converts to valproic acid in the body, appears to be related to the increase in GABA in the central nervous system. The doses indicated for epilepsy start at 10–15 mg/kg/day, and can be increased by 5–10 mg/kg/day weekly. Doses below 60 mg/kg/day are considered to have a good clinical response. For neuropathic pain, the literature suggests doses between 250 and 1500 mg/day, with a fractioned dose. In addition to the uses in chronic pain, there are reports of treatments with sodium valproate in patients with inflammatory verrucous epidermal nevi (ILVEN).^24^

### Topiramate (Amato®, Sigmax®)

An anticonvulsant medication, with multiple mechanisms to reduce neuronal hyperexcitability, used to treat epilepsy and migraine prophylaxis. It is recommended to start at a dose of 25–50 mg daily for at least one week, and increase the dose every one or two weeks. The daily dose should range between 200 and 400 mg/day and the maximum dose is 1600 mg.^11^, ^24^ There are reports of the use of topiramate in the treatment of trichotillomania, however studies with larger populations are necessary to confirm its effectiveness.^26^

### Pregabalin (Lyrica®)

This medication acts in the neuronal calcium channels. Its formal indication is for neuropathic pain, epilepsy, and fibromyalgia. Pregabalin has a good action in the control of neuropathic pain and has a good safety profile, because it interacts with fewer drugs when compared with other medications in this class. Some authors also indicate its use for uremic pruritus. A dose of 300–600 mg/day is indicated. Generally, it has no serious side effects; the literature reports side effects such as drowsiness, dizziness, and even peripheral edema.^11^, ^19^, ^24^

### Gabapentin (Gamibetal®, Progresse®, Gabaneurin®)

Generally used for epilepsy and neuropathic pain, gabapentin is an anticonvulsant derived from the neurotransmitter GABA. It is the most studied mood stabilizer in dermatology; it is especially effective in situations where sensitization of the central nervous system is a mediating factor. It is used more widely in post-herpetic neuralgia, but it can also be used in chronic pruritus and other pains of neural origin, such as paresthetic notalgia and brachioradial itching.^25^, ^27^, ^28^ It can be prescribed at a dosage of 300–3600 mg/day. This medication has a good safety standard regarding drug interactions and its most reported adverse effects are: drowsiness, nausea, double vision, and dysphasia, among others. There is insufficient data for use in pregnancy and lactation.^15^, ^29^

## Anxiolytics

Anxiolytic drugs are used to relieve anxiety symptoms, acting directly on the limbic system. Used in special situations, such as panic disorder and post-traumatic stress, for example, they have a high sedative power and may cause dependence. They are divided into benzodiazepines and non-benzodiazepines.^15^

### Benzodiazepines

Pharmacologically, benzodiazepines act as GABA modulators. The use of these drugs should be limited to a period of three to four weeks, due to the high potential for dependence and abuse. Some authors mention a maximum use of six weeks. Moreover, it is necessary to be careful when withdrawing the medication, due to the possibility of withdrawal symptoms^16^ They can be divided into short duration: triazolam (Halcion®), midazolam (Dormonid®); short-intermediate: alprazolam (Frontal®, Alfron®); intermediate-prolonged: bromazepam (Lexotan®), nitrazepam (Sonebon®), Lorazepam (Lorax); prolonged: clonazepam (Rivotril®), diazepam (Valium®), clobazam (Frisium®, Urbanil®), flurazepam (Dalmadorm®).

Among the adverse effects of these medications, learning difficulties, amnesia, aggressiveness, and confusion can be mentioned. There is a risk of respiratory depression in patients with chronic lung diseases, as well as with the use of central nervous system-depressant drugs (alcohol). They are contraindicated in the first trimester of pregnancy.^15^, ^17^

## Non-benzodiazepines

### Buspirone (Ansitec®)

Buspirone is a non-benzodiazepine anxiolytic drug that has no potential for dependence. Its onset of action is a bit slower, two to four weeks, and therefore it should not be used in more acute cases. Due to its profile, it is preferred in cases where longer therapies need to be instituted. The initial dose suggested is 15 mg/day, increasing 15 mg every week, with a maximum dose of 60 mg/day, divided into two doses, due to its short half-life.^11^, ^16^

### Zolpidem (Stilnox®)

Used as a sleep inducer, zolpidem should be used at a dose of 5–10 mg before bed. This drug acts in a similar way to benzodiazepines, and can lead to mental confusion and tolerance over time; therefore, it should be used for short periods.

## Adverse skin reactions to psychiatric drugs

Adverse skin reactions triggered by the use of psychotropic drugs are estimated depending on the drug in up to 39% of cases, with an increased risk associated with females, the elderly, and those of African descent.^30^ Among psychotropics, mood stabilizers present the highest prevalence and severity of skin reactions, which can be potentially fatal.^31^

The management of skin reactions associated with psychiatric drugs and the decision to discontinue treatment must be evaluated taking into account the severity of the skin manifestation and of the psychiatric illness. Moreover, the possible harm in relation to abrupt discontinuation of medication must be considered and evaluated.

### Pruritus^30^, ^31^, ^32^, ^33^, ^34^, ^35^, ^36^

Pruritus can occur with the use of any antidepressant, mood stabilizer, and antipsychotic. It is also reported by those using benzodiazepines, although less frequently.

Antidepressants: bupropion has the highest incidence of pruritus, while the lowest rates are found with fluoxetine, paroxetine, sertraline, and venlafaxine.

Mood stabilizers: all drugs in this class.

Antipsychotics: risperidone, olanzapine, quetiapine, and clozapine.

Benzodiazepines: there is a higher incidence for the use of alprazolam.

### Rash^30^, ^31^, ^32^, ^33^, ^34^, ^35^, ^36^

It is the most common adverse skin reaction triggered by the use of psychotropics. It is associated with the use of antidepressants, mood stabilizers, and antipsychotics.

Antidepressants: in all drugs in this class. Among tricyclic antidepressants, the highest incidence was observed with the use of clomipramine.

Mood stabilizers: carbamazepine, gabapentin, lithium, valproic acid, lamotrigine, and topiramate.

Antipsychotics: risperidone, olanzapine, quetiapine, clozapine, haloperidol, and ziprasidone.

Benzodiazepines: the highest incidence was observed with the use of alprazolam.

### Urticaria and angioedema^30^, ^31^, ^32^, ^33^, ^34^, ^35^, ^36^

They are common adverse manifestations. They are associated with the use of antidepressants, mood stabilizers, and antipsychotics.

Antidepressants: in all drugs in this class.

Mood stabilizers: carbamazepine and lamotrigine.

Antipsychotics: risperidone, olanzapine, clozapine, and haloperidol.

Benzodiazepines: alprazolam.

### Fixed drug eruption^30^, ^31^, ^32^, ^33^, ^34^, ^35^, ^36^

Lesions appear within eight hours after taking the medication, and are usually asymptomatic.

Antidepressants: in all drugs in this class.

Mood stabilizers: carbamazepine, lithium, gabapentin.

Antipsychotics: risperidone, olanzapine, quetiapine, and haloperidol.

### Photosensitivity^30^, ^31^, ^32^, ^33^, ^34^, ^35^, ^36^

Photosensitivity reactions (divided into phototoxic and photoallergic) are triggered by exposure to ultraviolet radiation with the use of certain medications.

Antidepressants: fluoxetine, paroxetine, sertraline, and escitalopram. There are few cases reported with the use of tricyclic antidepressants.

Mood stabilizers: carbamazepine and lamotrigine.

Antipsychotics: chlorpromazine, risperidone, olanzapine, quetiapine, clozapine, and haloperidol.

### Skin discoloration^30^, ^31^, ^32^, ^33^, ^34^, ^35^, ^36^

Skin discoloration usually occurs after prolonged use of some psychotropic drugs. In most cases, it disappears slowly after treatment discontinuation. It can take months or years for the pigmentation to completely disappear.

Antidepressants: the highest incidence is observed with tricyclic antidepressants.

Mood stabilizers: carbamazepine, gabapentin, and lamotrigine.

Antipsychotics: chlorpromazine, risperidone, olanzapine, quetiapine, clozapine, and haloperidol.

### Alopecia^30^, ^31^, ^32^, ^33^, ^34^, ^35^, ^36^

It usually occurs diffusely. Hair loss ceases with the discontinuation of medication. It is associated with the use of some antidepressants, mood stabilizers, and antipsychotics.

Antidepressants: selective serotonin reuptake inhibitors.

Mood stabilizers: lithium, carbamazepine, lamotrigine, and valproic acid.

Antipsychotics: risperidone, olanzapine.

### Acneiform rashes^30^, ^31^, ^32^, ^33^, ^34^, ^35^, ^36^

The lesions usually present as follicular pustules, without comedones. They occur on the face, chest, and upper back. They are mainly associated with the use of antidepressants.

Antidepressants: in all drugs in this class.

Mood stabilizers: lithium, carbamazepine, lamotrigine, and topiramate.

Antipsychotics: quetiapine and risperidone.

### Psoriasiform reactions^30^, ^31^, ^32^, ^33^, ^34^, ^35^, ^36^

The lesions are usually observed bilaterally on the elbows, knees, and scalp.

Antidepressants: fluoxetine, escitalopram, and venlafaxine.

Mood stabilizers: lithium, carbamazepine, and valproic acid.

Antipsychotics: risperidone and quetiapine.

### Seborrheic dermatitis^30^, ^31^, ^32^, ^33^, ^34^, ^35^, ^36^

It is a common manifestation of psychiatric drugs, especially due to the use of antidepressants and mood stabilizers.

Antidepressants: fluoxetine, paroxetine, and venlafaxine.

Mood stabilizers: lithium, carbamazepine, and valproic acid.

Antipsychotics: risperidone, olanzapine, clozapine, and haloperidol.

### Erythema multiforme^30^, ^31^, ^32^, ^33^, ^34^, ^35^, ^36^

It is a rare and serious adverse reaction. Cases of erythema multiforme-like lesions with the use of antidepressants and antipsychotics have been described in the literature.

Antidepressants: fluoxetine, paroxetine, sertraline, duloxetine, and bupropion.

Mood stabilizers: carbamazepine, lamotrigine, gabapentin, and valproic acid.

Antipsychotics: risperidone and clozapine.

### Stevens–Johnson syndrome and toxic epidermal necrolysis^30^, ^31^, ^32^, ^33^, ^34^, ^35^, ^36^

These are serious adverse reactions mainly associated with the use of mood stabilizers. They are rarely observed with the use of antidepressants. The involved medication should never be prescribed again.

Antidepressants: fluoxetine, sertraline, paroxetine, bupropion, and duloxetine.

Mood stabilizers: carbamazepine, lamotrigine, and valproic acid.

Antipsychotics: quetiapine and clozapine.

### Exfoliative erythroderma^30^, ^31^, ^32^, ^33^, ^34^, ^35^, ^36^

It is a serious adverse reaction. It is more associated with the use of some tricyclic antidepressants, and rarely occurs with the use of antipsychotics.

Antidepressants: amitriptyline, nortriptyline, clomipramine, and mirtazapine.

Mood stabilizers: lithium and carbamazepine.

Antipsychotics: risperidone and quetiapine.

### DRESS syndrome^30^, ^31^, ^32^, ^33^, ^34^, ^35^, ^36^

It is a serious adverse reaction; in addition to skin involvement, there is fever and involvement of several organs.

Antidepressants: amitriptyline, imipramine, and fluoxetine.

Mood stabilizers: carbamazepine, lamotrigine, and valproic acid.

Antipsychotics: olanzapine.

### Hypersensitivity vasculitis^30^, ^31^, ^32^, ^33^, ^34^, ^35^, ^36^

Initially, it is manifested by purpura in the lower limbs. There may be systemic involvement of different organs.

Antidepressants: paroxetine, fluoxetine, and sertraline.

Mood stabilizers: carbamazepine and lamotrigine.

Antipsychotics: clozapine and haloperidol.

## Conclusion

The identification and psychopharmacological management of psychocutaneous disorders should not be neglected by the dermatologist, since dermatological patients have a high prevalence of psychiatric comorbidities. The ability to prescribe psychotropic drugs becomes even more relevant when psychiatric treatment is neglected by the patient. Furthermore, the prescription of psychiatric drugs by a dermatologist (rather than a psychiatrist) may be better accepted, due to the stigma surrounding mental health and treatment with those professionals.

The dermatologist must be aware of the mechanisms, indications, and side effects of the most used psychotropic agents so that they can provide the best treatment and prevent the worsening of psychodermatoses. However, it is necessary to establish an attitude of empathy and support for these patients, so that they accept and adhere to the psychotropic intervention. The choice of the psychotropic drug must be based on the underlying psychopathology; depressive disorders, anxiety disorders, psychotic, delusional disorders, and obsessive–compulsive disorders are the most commonly found in dermatological practice.

It should also be noted that the use of psychotropic drugs is one of the components of a comprehensive treatment for patients with psychodermatoses. One should continue to encourage the search for psychiatric treatment and psychotherapeutic support so that the patient has better therapeutic results and a better quality of life.

## Financial support

None.

## Authors’ contributions

Magda Blessmann Weber: Conception and planning of the study; effective participation in research orientation; critical review of the literature; critical review of the manuscript.

Júlia Kanaan Recuero: Elaboration and writing of the manuscript; critical review of the literature; critical review of the manuscript.

Camila Saraiva Almeida: Preparation and writing of the manuscript; critical review of the literature; critical review of the manuscript.

## Conflicts of interest

None declared.

## Figures and Tables

**Table:** 

1. **Check the incorrect statement**:
a) Psychophysiological disorders include primary dermatoses that can be aggravated by emotional factors or stress.
b) Primary psychiatric disorders can lead to skin manifestations.
c) Secondary psychiatric disorders are not related to psychodermatoses.
d) Sensitive diseases of the skin or mucous membranes do not present primary dermatological lesions.
2. **Check the correct statement regarding antidepressants**:
a) Antidepressants have specific indications for certain dermatoses.
b) It may take up to two months for the desired therapeutic effects to be observed.
c) Tricyclic antidepressants present the greatest number of side effects.
d) Treatment can be withdrawn as soon as the desired therapeutic effects are achieved.
3. **Regarding SSRIs, it is correct to state that**:
a) They should not be used during pregnancy.
b) They have good tolerability and one of the main side effects is libido reduction.
c) Regular follow-up of patients is required, regardless of age.
d) Sertraline should not be used in patients with liver disease.
4. **For tricyclic antidepressants, check the incorrect statement**:
a) It is the oldest class of antidepressants.
b) The most relevant side effects are dry mouth, constipation, dizziness, tachycardia, and urinary retention.
c) Neuropathic pain and pruritus respond to treatment at doses lower than those generally used.
d) Patients with cardiovascular disease and those with a recent history of myocardial infarction can use the drug without restrictions.
5. **Check the correct statement regarding antipsychotics**:
a) They are divided between typical and atypical, and atypical antipsychotics are the newest.
b) They can be used in the elderly population without the need for specialized care.
c) They rarely cause adverse skin reactions.
d) They have little effect when used for the treatment of parasitic delirium and dermatitis artefacta.
6. **Regarding mood stabilizers, check the incorrect statement**:
a) They include anti-epileptics and lithium.
b) They are very effective in the treatment of neuropathic pain and pruritus.
c) They have no teratogenic effects and can be used without restriction during pregnancy.
d) They can also be used in psychodermatoses related to compulsion.
7. **Check the incorrect statement regarding benzodiazepines**:
a) They are addictive drugs and should not be used for a long time.
b) Rapid withdrawal of the drug can cause withdrawal symptoms.
c) Side effects such as amnesia, aggressiveness, and mental confusion can occur with the use of these drugs.
d) The can be used without restrictions in patients with chronic lung disease.
8. **Check the incorrect statement**:
a) Psychotropic drugs, in general, can cause unwanted skin reactions in patients treated with these drugs.
b) Common manifestations are pruritus, rash, and urticaria/angioedema.
c) The use of antidepressants of all classes may cause fixed drug eruptions.
d) Photosensitivity reactions are rare in all drugs used for psychodermatoses.
9. **Psoriasiform reactions and seborrheic dermatitis are more common with**:
a) Antidepressants and mood stabilizers
b) Benzodiazepines
c) Tricyclic antidepressants
d) Lithium
10. **SJS and TEN occur most frequently with which of the following medications**:
a) Antidepressants
b) Mood stabilizers
c) Antipsychotics
d) Benzodiazepines

**Table:** 

1. b	3. b	5. c	7. b	9. d
2. c	4. d	6. c	8. c	10. a

## CME Questions

ANSWERS

Update on parasitic dermatoses. An Bras Dermatol. 2020;95(1):1–14.
